# Zinc Sensing Receptor Signaling, Mediated by GPR39, Reduces Butyrate-Induced Cell Death in HT29 Colonocytes via Upregulation of Clusterin

**DOI:** 10.1371/journal.pone.0035482

**Published:** 2012-04-24

**Authors:** Limor Cohen, Hagit Azriel-Tamir, Natan Arotsker, Israel Sekler, Michal Hershfinkel

**Affiliations:** 1 Department of Morphology, Faculty of Health Science, Ben Gurion University of the Negev, Beer-Sheva, Israel; 2 Department of Physiology, Faculty of Health Science, Ben Gurion University of the Negev, Beer-Sheva, Israel; Queen Mary University of London, United Kingdom

## Abstract

Zinc enhances epithelial proliferation, protects the digestive epithelial layer and has profound antiulcerative and antidiarrheal roles in the colon. Despite the clinical significance of this ion, the mechanisms linking zinc to these cellular processes are poorly understood. We have previously identified an extracellular Zn^2+^ sensing G-protein coupled receptor (ZnR) that activates Ca^2+^ signaling in colonocytes, but its molecular identity as well as its effects on colonocytes' survival remained elusive. Here, we show that Zn^2+^, by activation of the ZnR, protects HT29 colonocytes from butyrate induced cell death. Silencing of the G-protein coupled receptor GPR39 expression abolished ZnR-dependent Ca^2+^ release and Zn^2+^-dependent survival of butyrate-treated colonocytes. Importantly, GPR39 also mediated ZnR-dependent upregulation of Na^+^/H^+^ exchange activity as this activity was found in native colon tissue but not in tissue obtained from GPR39 knock-out mice. Although ZnR-dependent upregulation of Na^+^/H^+^ exchange reduced the cellular acid load induced by butyrate, it did not rescue HT29 cells from butyrate induced cell death. ZnR/GPR39 activation however, increased the expression of the anti-apoptotic protein clusterin in butyrate-treated cells. Furthermore, silencing of clusterin abolished the Zn^2+^-dependent survival of HT29 cells. Altogether, our results demonstrate that extracellular Zn^2+^, acting through ZnR, regulates intracellular pH and clusterin expression thereby enhancing survival of HT29 colonocytes. Moreover, we identify GPR39 as the molecular moiety of ZnR in HT29 and native colonocytes.

## Introduction

Colonocytes are constantly exposed to short chain fatty acids (SCFA), produced in the colonic lumen by bacterial fermentation of carbohydrates and dietary fibers [1]. Most prominent among these SCFAs is butyrate, an important energy source for colonocytes that is also regulating physiological functions such as transepithelial ion and electrolyte transport [2], [3]. Butyrate further regulates differentiation, proliferation and cell survival of colonocytes, however these effects depend on the differentiation level of the cells [4], [5]. Consequently, butyrate selectively induces growth inhibition and differentiation subsequently leading to apoptosis in a variety of human colon cancer cell lines [6], [7], [8], [9], but not in normal colonocytes. Although its precise mechanism of action is unclear, butyrate effectively regulates the expression of an array of proteins leading to apoptosis of colon cancer cells [9], [10], [11], [12]. Consistently, an inverse relationship is found between the level of dietary fibers and the incidence of human colon cancer, and it has been suggested that butyrate reduces foci number and size [8], [13], [14]. In addition, changes in expression of butyrate transporters and receptors in human cancer tissue further support a role for this SCFA in colon cancer [15], [16], [17], [18].

Zinc is an essential micronutrient that has an established role in enhancing epithelial proliferation and survival [19], [20], [21], [22], moreover, zinc signaling is suggested to play a role in cancer [23], [24]. Zinc has been specifically shown to play a role in digestive system function under normal conditions or stress [25], [26], [27]. Furthermore, zinc accelerates healing of gastric ulcers and drugs containing zinc are commonly used to enhance colonocytes cell proliferation and growth, although the mechanism is unknown [25], [28]. Similarly, Zn^2+^ has been suggested to protect cells against butyrate induced cell death but the cellular pathways underlying this effect are not understood [20]. We previously identified a Zn^2+^-sensing receptor (ZnR) that is a G-protein coupled receptor. The ZnR complexes to Gαq subunits which activate phospholipaseC β (PLCβ). This in turn induces formation of IP_3_ leading to release of intracellular Ca^2+^. Using dose response analysis we showed that ZnR is activated by Zn^2+^ concentration that is physiologically relevant in the colon, suggesting that this receptor may be regulating the function of colonocytes [29]. In HT29 colonocytes and PC-3 prostate cancer cells, the ZnR mediates Zn^2+^-dependent activation of the MAP and PI3 kinase pathways [21], [22], [30]. Desensitization of ZnR by zinc is followed by inhibition of the Zn^2+^-dependent signaling as well as proliferation and survival of prostate cancer cells [21]. Furthermore, activation of ZnR signaling upregulates the Na^+^/H^+^ exchanger (NHE) and enhances recovery from acidic pH [22]. The molecular identity of the ZnR remained elusive until G protein-coupled receptor 39 (GPR39), previously thought to interact with obestatin, was shown to interact with Zn^2+^ when ectopically expressed [31], [32]. Remarkably, GPR39 is expressed in the gastrointestinal tract [31] and in the brain where ZnR activity was identified [33]. However whether GPR39 mediates Zn^2+^-dependent signaling in the colon [22], [34] is unknown. Here, we find that GPR39 is the receptor that mediates ZnR-signaling in colonocytes and elucidate its physiological role in promoting survival of coloncytes. Our results further indicate that extracellular Zn^2+^, via GPR39 signaling, confers resistance against butyrate induced cell death in HT29 cells.

## Methods

### Cell Culture

HT29-Cl cells (obtained from American Type Culture Collection) were grown in DMEM medium (Sigma-Aldrich, USA) containing, 100 U/ml penicillin, 0.1 mg/ml streptomycin, 2 mM glutamine and 10% fetal calf serum (Biological Industries, Israel), in a 5% CO_2_ humidified atmosphere at 37°C.

### GPR39 silencing

HT29 cells were seeded in 60-mm cell culture dishes at a density of 7×10^5^ cells or in 96-well plates (1.5×10^4^ cells) 24 h prior to transfection in standard DMEM culture media as described above, but without penicillin or streptomycin. Cells were transfected using Lipofectamine 2000 according to the manufacturer protocol, and used for the different experimental settings 48 h post transfection. For silencing of target proteins siRNA constructs (Sigma-Aldrich, Israel) were utilized, for GPR39: 5′-CCA UGG AGU UCU ACA GCA U-3′, for CLU: 5′-CCA GAG CUC GCC CUU CUA C-3′ and siRNA control (scrambled) sequence was 5′- GCC CAG AUC CCU GUA CGU-3′.

### SRB assay for cell density

Sulforhodamine B (SRB) assay was used to assess cell survival of HT29 cells [35]. HT29 cells were seeded into 96-well plates (30,000 cells per well) and serum-starved for 24 h. For ZnR activation, cells were transferred into Ringer's solution and treated with Zn^2+^ (80 µM, 10 min) or without it (as control) and then returned to serum-free growth media. This procedure was repeated during 3 consecutive days, as previously described [21]. Butyrate (30 mM, pH7.4, for 24 h) was applied following the first application of Zn^2+^, and then cells were returned to serum-free growth media. Cells were fixed after 96 hours using 10% TCA (trichloroacetic acid) for 1 hour at 4°C. The supernatant was discarded and plates were washed with deionized water and air-dried. 100 µl sulforhodamine B (SRB 0.4 w/v in 1% acetic acid) was added to each well and the culture was incubated for 10 min at room temperature. The unbound SRB was removed by washing with 1% acetic acid and the plates were air-dried. The dye bound to basic amino acids of the cell membrane was solubilized with Tris buffer (10 mM, pH 10.5) and the absorption measured at 540 nm by ELISA reader (Molecular Devices) to determine the relative cell growth or viability in the treated as well as untreated cells. Cell number was quantified using a calibration curve and cell survival was determined as percentage of the number monitored in control cells (non-treated, 100%). Each graph represents an average of at least three independent experiments.

**Real Time PCR** – Real time PCR was used to estimate the GPR39 silencing. Cells were seeded on a 60-mm plates, after 48 hours cells were trypsinized with 0.05% trypsin (Biological Industries, Kibbutz Beit Haemek, Israel), collected. Cell lysates were homogenized using QIAshredder as described by the manufacturer (QIAGEN). Total RNA was purified using RNeasy Mini Kit as described by the manufacturer (QIAGEN). 1 µg RNA was converted to cDNA using Verso cDNA synthesis Kit as described by the manufacturer (Thermo Scientific). cDNA was diluted 1∶16 (dilution was chosen after calibration) with ultrapure water, and subjected to real time PCR procedure (Taqmen), which was done with ABsolute Blue QPCR kit as described by the manufacturer (Thermo Scientific). Primers and probes were supplied by Solaris by the following sequences: for GPR39: forward primer CATCTTCCTGAGGCTGA, reverse primer ATGATCCTCCGTCTGGTTG, probe TATGCTGGATGCCCAAC, and for Actin: forward primer TGGAGAAAATCTGGC-ACCAC, reverse primer GGTCTCAAACA TGATCTGG, probe ACCGCCAGAAGATGACC.

### Monitoring clusterin expression

HT29 cells were seeded on 60-mm plates (1.4×10^6^ cells), and serum-starved for 24 hours. Cells were then treated daily with Zn^2+^ (80 µM for 10 min) in Ringer's solution, in the presence or absence of the indicated inhibitors, added 30 min prior to Zn^2+^ treatment. Butyrate (30 mM, pH 7.4 in serum-free medium) was added after the first Zn^2+^ application for 24 h. Cells were harvested following 96 h into lysis buffer (50 mM HEPES, pH 7.5, 150 mM NaCl, 1 mM EDTA, 1 mM EGTA, 10% glycerol, 1% Triton X-100, 10 µM MgCl2, 20 mM *p*-nitrophenyl phosphate, 1 mM Na3VO4, 25 mM NaF), in the presence of Protease Inhibitor Cocktail (1∶25 Complete, Roche, Germany). Lysates were placed on ice for 10 min and then centrifuged for 30 min (14,000 rpm) at 4°C. Supernatants (cytosolic fraction) were collected, protein concentrations were determined using Bio-Rad protein assay, SDS sample buffer was added, and samples were boiled for 5 min and then frozen at −80°C until used. Whole cell lysates (50 µg) were separated on 8–10% SDS–PAGE and blotted onto nitrocellulose membranes. The required proteins were detected using specific antibodies raised against, α-Clusterin (Santa-Cruz, USA) or β-actin (Cell Signaling, USA). Densitometric analysis of expression level was performed using the EZQuant-Gel image processing and analysis software (EZQuant, Rehovot, Israel). Clusterin levels were normalized to actin levels, and are presented as a percentage of the maximal expression level induced by application of Zn^2+^ and butyrate. Each graph represents an average of at least three independent experiments.

### Monitoring Kinase Activation

HT29 cells were seeded on 60-mm culture dishes and serum starved as described above. Cells were Zn^2+^ treated (80 µM for 10 min) in Ringer's solution, in the presence or absence of the indicated inhibitors, added 30 min prior to Zn^2+^ treatment (except LY294002, added 60 min prior to Zn^2+^ treatment), and incubated for an additional 10 min after the Zn^2+^ treatment to allow kinase phosphorylation. Next, cells were harvested as described above, whole cell lysates (50 µg) were separated on 10% SDS-PAGE and blotted onto nitrocellulose membranes. The required proteins were detected using specific antibodies raised against the doubly phosphorylated ERK1/2 and total ERK1/2 or phosphorylated AKT and total AKT. Densitometric analysis of expression level was performed as described above. Phospho-ERK1/2 or AKT levels were normalized against the total ERK1/2 or AKT protein, respectively. Phosphorylation of ERK1/2 or AKT is presented as a percentage of the effect triggered by application of 80 µM Zn^2+^. Each graph represents an average of at least three independent experiments.

### Fluorescent Imaging

The imaging system consisted of an Axiovert 100 inverted microscope (Zeiss), Polychrome V monochromator (TILL Photonics, Germany) and a SensiCam cooled charge-coupled device (PCO, Germany). Fluorescent imaging measurements were acquired with Imaging Workbench 5 (Indec, CA). All results shown are representative traces from one slide showing the averaged responses of 15–25 cells. Bar graphs exhibit the mean response of n slides (as indicated in the figure legend) taken from at least 3 independent experiments.

#### Calcium imaging

For [Ca^2+^]i measurements, cells were incubated for 30 min with 2.5 µM Fura-2 acetoxymethyl ester (AM; TEF-Lab, USA) in Ringer's solution with 0.1% BSA. After dye loading, the cells were washed in Ringer's solution, and the cover slides were mounted in a chamber that allowed the superfusion of cells. Fura-2 was excited at 340 nm and 380 nm and imaged with a 510 nm long-pass filter.

#### pH Imaging

By using the same experimental setup, HT29 cells were loaded for 12 min with 1.25 µM 2,7-bis-(carboxyethyl)-5-(and-6)-carboxyfluorescein (BCECF)-AM (TEF-Lab, USA) in 0.1% BSA in Ringer's solution. The excitation was at 440 nm and 480 nm, and emission was monitored through a 510 nm long-pass filter. For pHi calibration, nigericin was added to KCl Ringer's (120 mM KCl replacing NaCl) solution at pH 6.8, 7 and 7.2, the relative fluorescence was monitored, and a linear calibration curve produced [36].

The NH_4_Cl prepulse paradigm was applied to estimate Na^+^/H^+^ exchanger activity. Briefly, cells were washed with Ringer's containing NH_4_Cl (30 mM), and an intracellular equilibrium of NH_4_^+^ and NH_3_ was reached leading to intracellular alkalinization. Replacing the extracellular buffer with Na^+^-free Ringer's (iso-osmotically replaced by NMG) caused rapid acidification of the cells ad the recovery rate from this acid load following addition of Na^+^ was monitored. For monitoring cellular acidification with butyric acid the following paradigm was employed: cells were washed with Na^+^-free Ringer's (iso-osmotically replaced by NMG) containing butyric acid (20 mM, pH 7.4), which caused rapid acidification. Replacing the extracellular buffer with Na^+^-containing Ringer's resulted in pHi recovery. The Na^+^/H^+^ exchanger activity was estimated by calculating the rates of recovery (dpHi/dt) following addition of Na^+^ to the Ringer's solution in both paradigms.

### Colon tissue preparation (including Ethics Statement)

GPR39^+/+^ (WT) or GPR39^−/−^ (KO) littermates [37] were used after genotyping using PCR of DNA isolated from mouse tail biopsy samples. Primers 5′- ACCCTCATCTTGGTGTACCT-3′ and 5′-TGTAGCGCTCAAAGCTGAG-3′ amplified a 311-bp band from the wild-type allele whereas primers 5′-GGAACTCTCACTCGACCTGGG-3′ and 5′-GCAGCGCATCGCCTTCTATC-3′ amplified a 262-bp band from the knockout allele. Colon tissue was prepared as described previously [22], [38] in accordance with protocols approved by the committee for the Ethical Care and Use of Animals in Research at Ben Gurion University. Briefly, male mice at ages of 2–4 weeks, were sacrificed and the colon removed and washed with a syringe using Parson's Solution. A longitudinal incision was made along the colon wall and the tissue was immersed in the solution. Tissue samples were cut from the distal part of the colon and were spread, keeping the mucosal-luminal side upwards, on coverslips using cyanoacrylate glue. Tissue samples were maintained at 37°C in high K^+^ solution for no longer than 3 h.

For the analysis of NHE activity in colon tissue the same paradigm that was used for the HT29 cells was employed. Loading of the tissue sections was performed in high K^+^ solution [38] containing 5 µM BCECF, 0.02% pluronic acid, 250 µM probenecid and 0.1% BSA. Subsequently the tissue was washed in Ringer's solution containing 0.1% BSA and imaging of intracellular pH was monitored. Since the tissue was more sensitive to phototoxicity induced during the imaging, in the initial phase of the experiment images were acquired every 10 s, while before the addition of Na^+^ the rate of acquisition was changed and an image was acquired every 3 s. In each experimental group, the results are the mean of at least 15 regions of interest from more than three independent experiments from each animal. The rate of NHE activity was calculated using a linear fit to the graph at the initial 50 s of this phase. The pHi calibration was performed using nigericin as described above at pH of 6.6, 7, 7.5 and 8.2.

### Statistical Analysis

Data are expressed as means ± SEM. Each treatment was compared with the control or Zn^2+^ treatment, and statistical significance between the groups was evaluated using the Student's *t* test or ANOVA followed by Tukey-Kramers test, as appropriate. *, *p*<0.05; **, *p*<0.01.

## Results

### Zn^2+^ enhances cell survival following exposure to butyrate

**Figure 1:**
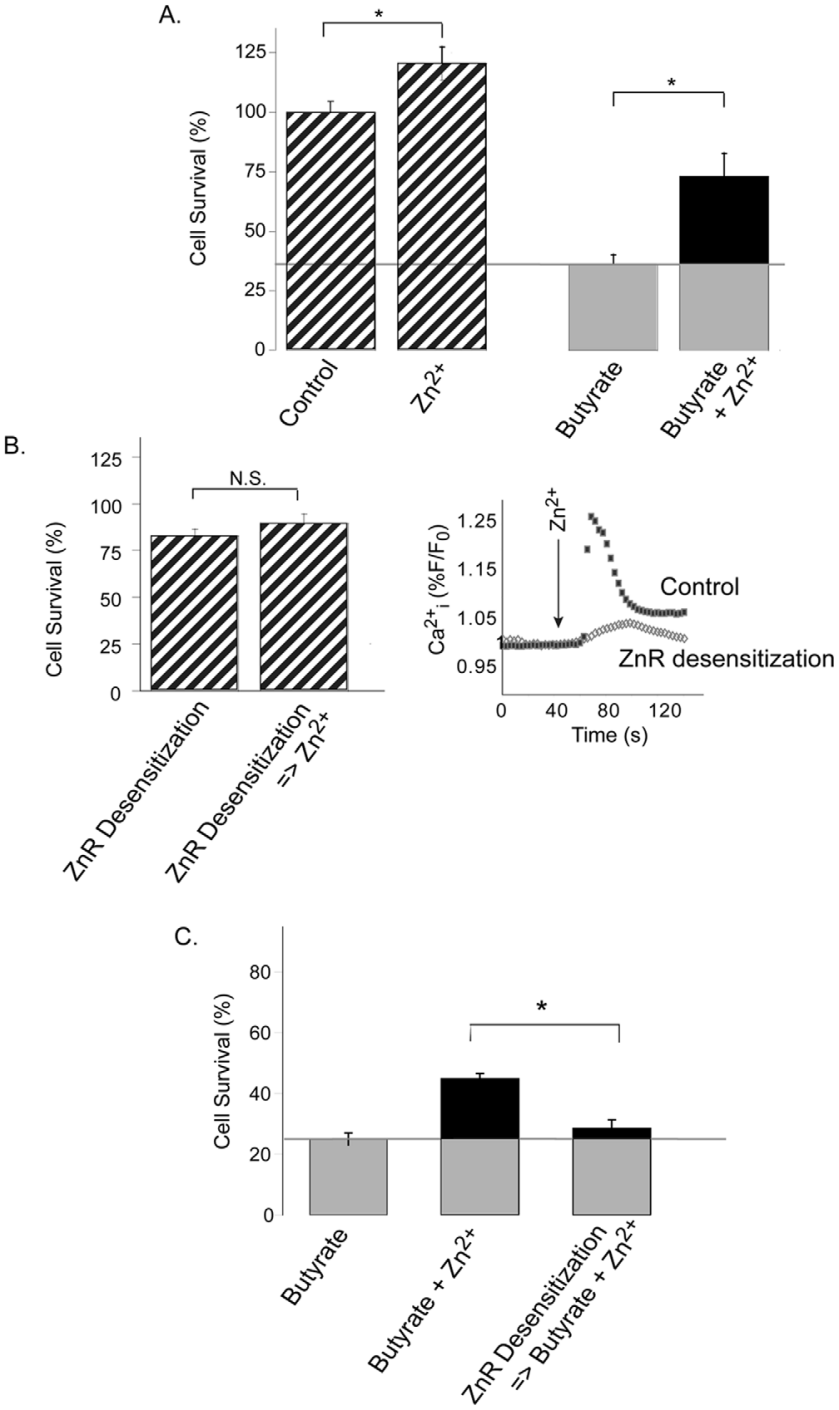
Extracellular Zn^2+^ reduces butyrate induced cell death and requires a functional ZnR. **A.** Cell numbers in cultures treated with butyrate (30 mM, 24 h) were compared to control cultures (without butyrate, hatched bars) using the SRB colorimetric assay. Cells were treated daily with Ringer's solution (10 min) without (control) or with Zn^2+^ (80 µM Zn^2+^) n = 6, *p<0.05. **B.** The effect of Zn^2+^ desensitization of ZnR (100 µM Zn^2+^, 15 min) on cell growth was determined. 30 min following ZnR desensitization, Zn^2+^ was re-applied to activate the ZnR as in A, or desensitized-cells were treated with Ringer's solution, and subsequently cell numbers were determined using the SRB assay. Following ZnR desensitization Zn^2+^ did not enhance cell numbers significantly. Similarly, Ca^2+^ release, monitored using Fura-2 fluorescence, in response to the re-application of Zn^2+^ was almost absent following desensitization of the ZnR (right panel). **C.** The effect of Zn^2+^ on cell survival was determined following desensitization of ZnR (100 µM Zn^2+^, 15 min) or in controls (Ringer's solution, 15 min), using the SRB assay. Cells were treated with butyrate and Zn^2+^ was re-applied to control cultures or cultures previously treated for ZnR desensitization (as indicated, see Methods). n = 5, *p<0.05.

We first asked if Zn^2+^ can enhance survival of HT29 cells exposed to the SCFA butyrate, known to trigger apoptotic signaling in colon cancer cells [5]. The ZnR in HT29 cells was briefly activated daily (80 µM Zn^2+^, 10 min) during 3 consecutive days, as previously described [21], and butyrate (30 mM, pH7.4, for 24 h) was applied following the first application of Zn^2+^. Cells were then fixed and the number of cells was determined using the SRB colorimetric assay (Fig. 1A). To determine if Zn^2+^ enhances cell growth we initially applied Zn^2+^ to control cells not treated with butyrate. Cell numbers were significantly higher (121±7% of the control cells) following the brief Zn^2+^ treatment suggesting that ZnR activation may enhance cell growth. Exposure of HT29 cells to butyrate resulted in survival of only 36±8% of the cells compared to untreated cells, consistent with previous studies [39], [40]. However, 63±11% of the cells survived when cells were pre-treated with Zn^2+^ (p<0.05 compared to butyrate alone, Fig. 1A).

In order evaluate the involvement of ZnR in the enhanced Zn^2+^-dependent survival of HT29 cells, we used functional desensitization of this receptor [22]. A procedure which does not result in Zn^2+^ permeation into the cells or in depletion of the Ca^2+^ stores [22]. Cells were treated with 100 µM Zn^2+^ for 15 min and washed in Ringer's solution for 30 min (desensitization protocol), subsequent application of Zn^2+^ did not induce the ZnR-dependent Ca^2+^ response in HT29 cells (right panel, Fig. 1B). Using the SRB assay we first compared cell numbers between 1) cultures that were treated with a brief application of Zn^2+^ (such as used for ZnR activation) subsequent to the desensitization procedure and 2) cells that were desensitized and not re-activated by Zn^2+^ (Fig. 1B, left panel). Cell numbers were reduced following ZnR desensitization compared to control cells which did not undergo the desensitization protocol (approximately 75% of controls, Fig. 1B). This suggests that ZnR activation, probably by residual Zn^2+^ in the growth medium, may be essential for cell growth. In contrast to the effect of Zn^2+^ on the control cells (Fig. 1A), following ZnR-desensitization re-application of Zn^2+^ did not change the number of cells, suggesting that ZnR signaling is essential to mediate Zn^2+^-dependent cell growth. We then asked if ZnR activation would also rescue cells from butyrate induced cell death. Functional ZnR desensitization was done prior to the daily activation of the ZnR, and 24 h of butyrate treatment followed the first application of Zn^2+^ (as in A). As control, cells were maintained in Zn^2+^-free Ringer's solution for the same time as the desensitized cells. Following ZnR desensitization, numbers of cells treated with butyrate only (25±2%, Fig. 1C) were similar to those treated with butyrate and Zn^2+^ (28±2%). Importantly, in the non-desensitized cells Zn^2+^ was still able to enhance cell survival (42±1%, p<0.05, Fig. 1C). Thus, desensitization and loss of ZnR activity eliminated the pro-survival effect of Zn^2+^ on butyrate-treated HT29 cells.

**Figure 2:**
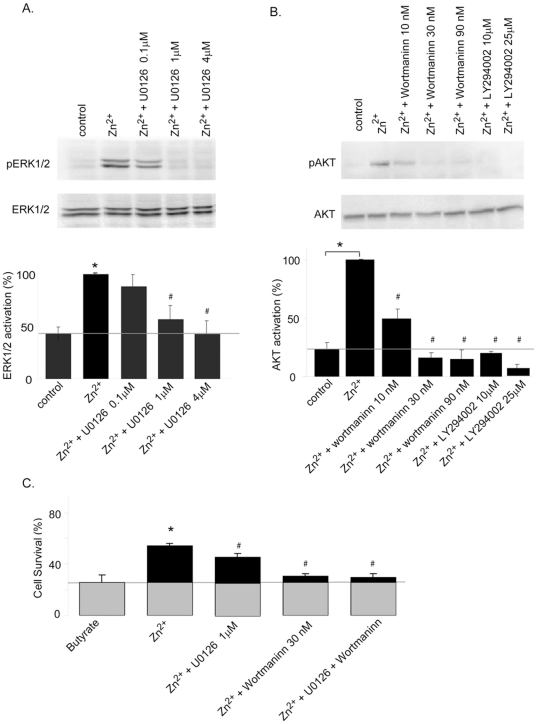
Inhibition of ZnR signaling reverses the pro-survival effects of Zn^2+^. **A–B.** HT29 cells were treated with Zn^2+^ for 10 min in Ringer's solution following 30–60 min incubation with the indicated concentrations of the MEK1/2 inhibitor U0126 or the PI3 kinase inhibitors wortmanin and LY294002. Cell lysates were immunoblotted with an anti ERK1/2 or p-ERK1/2 (A), and with an anti-AKT or p-AKT (B). Desnitometry analyses are presented, *bottom panels*, the results are normalized to the maximal activation obtained by the Zn^2+^ treatment. n = 3 *p<0.05 compared to control and #p<0.05 compared to Zn^2+^ treated cells. **C.** Cell numbers were determined in cells treated with butyrate and Zn^2+^ in the presence or absence of U0126 (1 µM), wortmannin (30 nM) or both. n = 3 * p<0.05 compared to control and #p<0.05 compared to Zn^2+^ treated cells.

We then sought to determine if ZnR-dependent activation of the PI3 and MAP kinase signaling is involved in enhancing cell survival. We assessed Zn^2+^-dependent ERK1/2 and AKT phosphorylation in the presence of the MEK1/2 inhibitor U0126, and the PI3K inhibitors wortmannin or LY294002 (Fig. 2A–B). We then studied if the effect of Zn^2+^ on cell survival is reversed by the inhibition of these pathways, using the lowest concentration which effectively abolished ZnR-dependent Ca^2+^ responses (Fig. 2C). The MEK1/2 inhibitor (U0126, 1 µM) somewhat reversed the effect of Zn^2+^ on cell survival, while PI3 kinase inhibition (wortmannin, 30 nM) largely eliminated the pro-survival effect of Zn^2+^ on HT29 cells exposed to butyrate. The addition of both inhibitors resulted in a survival rate similar to that of the cells treated with wortmannin alone. This indicates that Zn^2+^-dependent activation of ZnR and its downstream signaling, largely via the PI3K, mediates the protective effects of Zn^2+^ from butyrate induced cell death.

### ZnR/GPR39 mediates Zn^2+^-dependent cell growth

**Figure 3:**
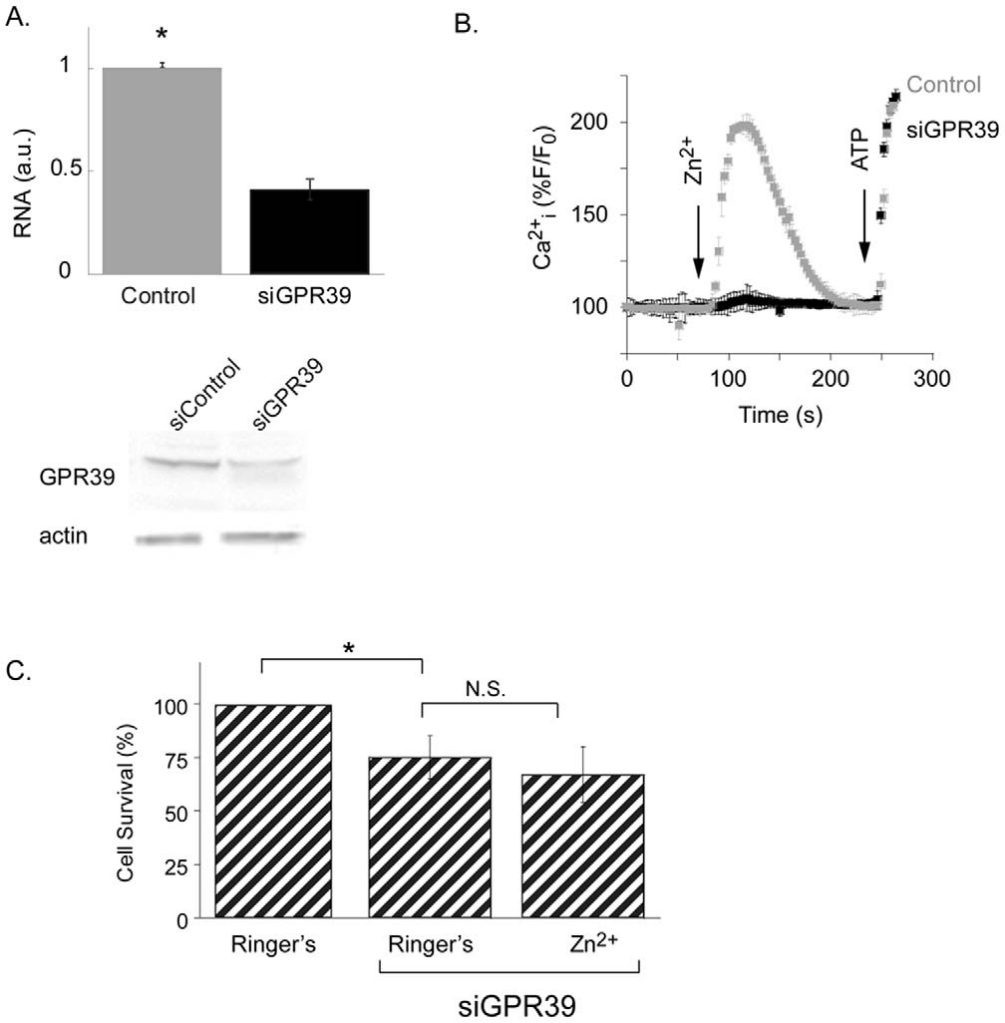
GPR39 mediates ZnR signaling and Zn^2+^- dependent cell growth. **A.** Cells were transfected with siRNA sequences compatible to GPR39 (siGPR39) or a scrambled sequence (siControl), and the mRNA and protein expression levels of GPR39 were monitored using Real-Time PCR (*top panel*) and western-blot analysis (*bottom panel*). **B.** The Zn^2+^ -dependent Ca^2+^_i_ responses were monitored in cells transfected with siGPR39 or siControl constructs using Fura-2 AM. ATP (50 µM) was subsequently applied to determine the integrity of the IP3 pathway. **C.** Cell numbers were determined using the SRB method in cultures transfected with siGPR39 or controls, which were treated with or without Zn^2+^ as in Figure 1A. n = 3 *p<0.05.

GPR39, a putative candidate of ZnR, is expressed in several tissues including the digestive tract. We therefore studied the effect of GPR39 silencing on Zn^2+^-dependent intracellular Ca^2+^ release. Transfection of HT29 cells with an siRNA construct designed to silence GPR39 (siGPR39) significantly reduced GPR39 RNA level and protein expression (Fig. 3A), followed by a dramatic reduction of Zn^2+^-dependent Ca^2+^ rise (Fig. 3B). ATP-dependent Ca^2+^ release in cells transfected with the siGPR39 or siControl constructs were similar, indicating that the IP3 pathway is not altered by GPR39 silencing. Next, we studied the effect of silencing GPR39 expression on HT29 cell growth. Interestingly, silencing GPR39 resulted in a slight decrease in cell numbers compared to control cells (Fig. 3C). This ZnR/GPR39-dependent cell growth is similar to that observed in the ZnR desensitized cells (Fig. 1B). In contrast to the effect of Zn^2+^ that enhanced HT29 cell numbers in control cells (121±7% of the control non-treated cells, Fig. 1A), cell numbers were similar in the siGPR39-transfected cells treated with Zn^2+^ (67±10%) or without it (75±13%, Fig. 3C). Thus, the results obtained in this set of experiments indicate that GPR39 is mediating ZnR signaling in HT29 colonocytes and that GPR39/ZnR signaling is required for normal cell growth.

### GPR39 mediates ZnR signaling in colon epithelium

**Figure 4:**
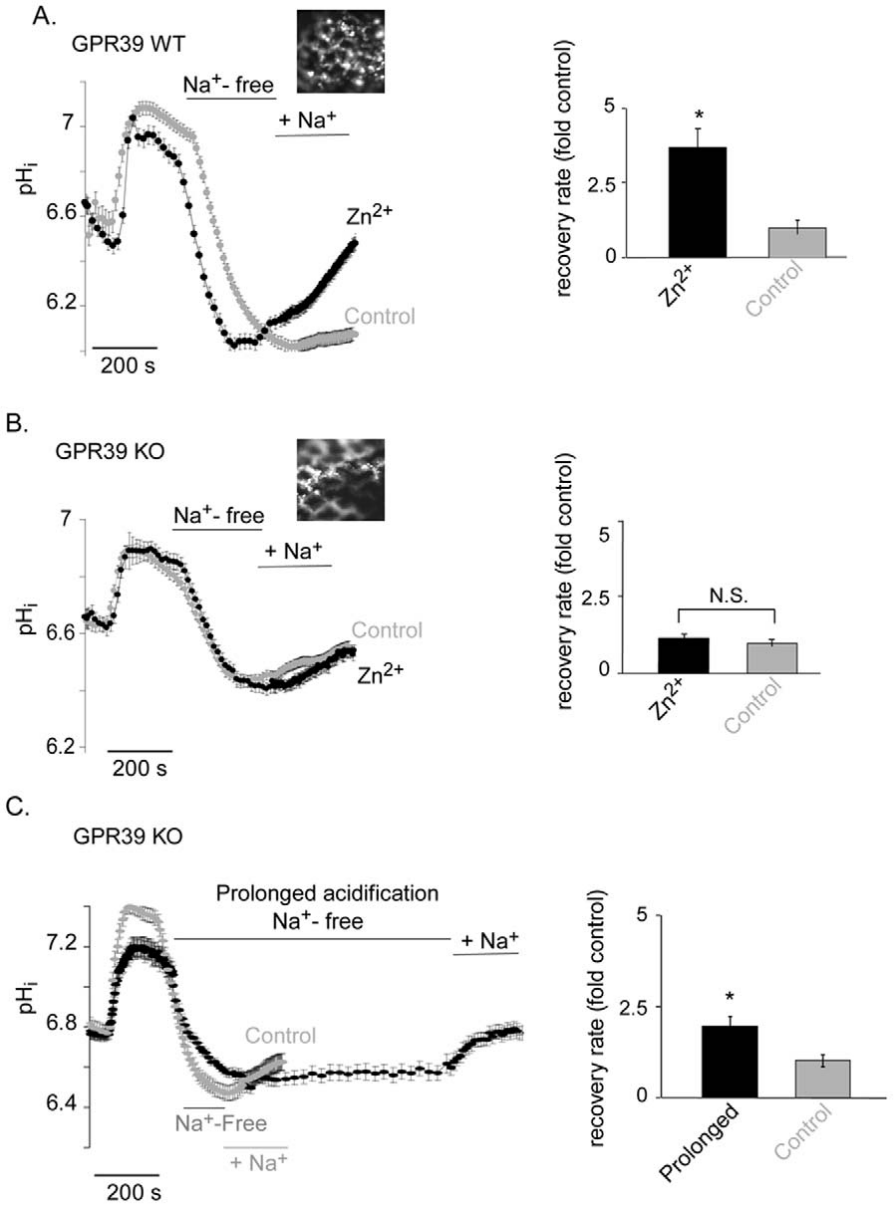
Zn^2+^-dependent activation of NHE in the colon is mediated by GPR39. **A–B.** Colon epithelium was obtained from WT and GPR39 KO mice as described in Materials and Methods. Colonocytes were loaded with the intracellular pH-sensitive dye (BCECF, 5 µM) inset was imaged at 440 nm using a 10× objective, showing the cells surrounding crypts are loaded with the dye. The NH_4_Cl prepulse paradigm was employed to determine NHE activity. Representative traces of the experiments from WT (A) and KO (B) tissues are shown in the *left panels*. In the *right panels*, the bar graph represents the average rate of Na^+^-dependent H^+^ efflux following acidification as calculated from the traces, and the effect of Zn^2+^ is presented as a fold increase of the basal NHE activity (control). n = 5, *p<0.05. **C.** The rate of Na^+^-dependent H^+^ efflux following acidification was monitored in GPR39 KO tissues exposed to short acidification period (control) or a prolonged acidification (by maintaining a Na^+^-free Ringer's solution for 10 min following initial acidification) and is presented as a fold increase of the Na/H exchange rate obtained in the short acidification. Representative traces are shown in the *left panel*. Na^+^/H^+^ exchange activity was determined as the rate of pHi recovery, averaged rates are presented in *right panel*. n = 3, *p<0.05.

To identify the role of GPR39 in mediating Zn^2+^-dependent signaling in colonocytes we employed colon tissue from GPR39 KO mice. We have previously monitored Zn^2+^-dependent upregulation of Na^+^/H^+^ exchange (NHE) activity, which is involved in recovery from acid load in colonocytes, but did not determine the role of ZnR in mediating this effect [22]. While essential for homeostatic cell maintenance, NHE activity may also be relevant to survival of colonocytes from butyrate induced cell death [41]. Mouse colon tissue was removed quickly and the tissue was spread and glued with the epithelial side exposed and immersed in high K^+^ solution [22]. The tissue was loaded with BCECF and mounted on a microscope holder allowing identification of cells surrounding the crypts, low magnification and high binning were used to minimize bleaching of the dye (inset Fig. 4B). The NH_4_Cl paradigm was applied and pH_i_ recovery rates following intracellular acidification were determined (see Materials and Methods). The rate of pHi recovery from the acid load, representing NHE activity, was compared between Zn^2+^ pre-treated (100 µM, 2 min) and control (Zn^2+^-free buffer) colon tissue. Similar to the previously reported effect of Zn^2+^ on NHE activation, the rate of pHi recovery was enhanced by 3.7±0.6 fold following Zn^2+^ treatment in the GPR39 WT tissue (Fig. 4A). To specifically address the role of GPR39 we compared the effect of Zn^2+^ on pH recovery in colon tissue obtained from GPR39 KO mice. Application of Zn^2+^ to GPR39 KO tissues did not enhance the recovery rate compared to the controls (Fig. 4B).

The recovery rate from acid load in control (without Zn^2+^) GPR39 KO tissue was faster than that observed in the GPR39 WT tissue, likely due to a compensatory mechanism. Hence we asked whether the recovery rate can be further upregulated in GPR39 KO tissues. We have previously shown that prolonged intracellular acidification activates NHE in HT29 cells, independent of Zn^2+^ treatment [22]. We therefore employed this paradigm in colon tissue obtained from GPR39 KO mice. Tissue samples loaded with BCECF were treated with NH_4_Cl as in Figure 4A, and then washed with Na^+^-free Ringer's solution. When the cells reached the acidic pH, instead of washing them with Na^+^-containing Ringer's solution, they were maintained for 10 more minutes with Na^+^-free Ringer's solution thereby achieving prolonged intracellular acidification. Then Na^+^-containing solution was applied and the pHi recovery rates were determined (Fig. 4C). The prolonged acidification was followed by increased recovery rate in the GPR39 KO tissues (2±0.2 fold compared to controls), suggesting the exchanger activity is not saturated in the knock-out tissue. Taken together with the fact that Zn^2+^ did not upregulate NHE activity in GPR39 KO colon tissue (Fig. 4B), this supports our conclusion that ZnR/GPR39 is essential for Zn^2+^-dependent regulation of NHE activity.

### The Na^+^/H^+^ exchanger mediates pHi recovery but not survival following application of butyrate

**Figure 5:**
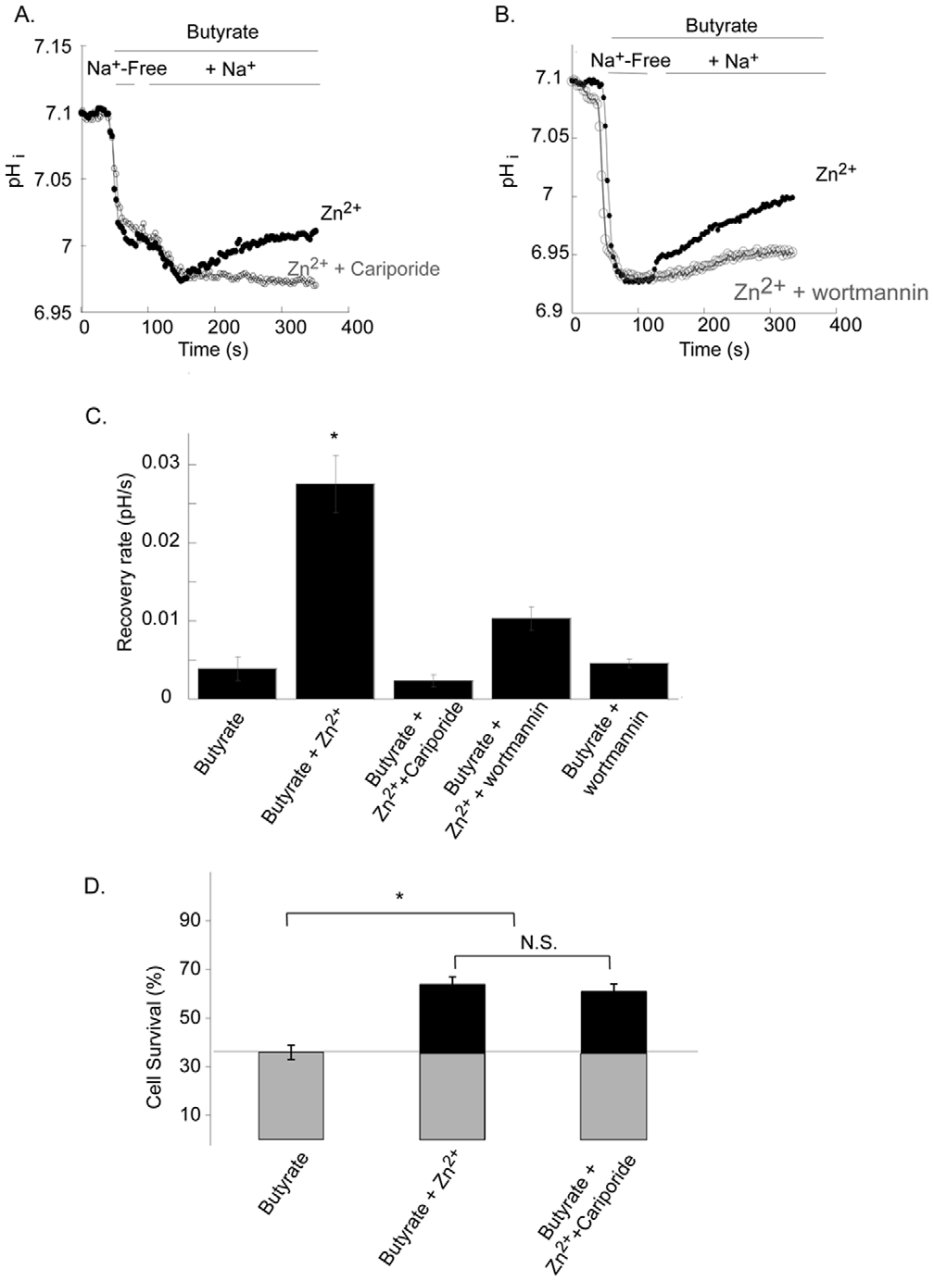
Zn^2+^-dependent pHi recovery following butyrate induced acid load involves PI3K signaling, and activation of NHE1 isoform, but does not contribute to cell survival. **A.** The pH sensitive dye BCECF was used to monitor pH_i_ in HT29 control cells, cells pretreated with Zn^2+^ (80 µM, 2 min) or cells treated with Zn^2+^ and the NHE1 inhibitor cariporide (0.5 µM). Cells were superfused with 30 mM butyrate (pH 7.4) in Na^+^-free Ringer's solution, and then Na^+^ was added to the Ringer's solution. Representative traces are shown. **B.** pHi was monitored following application of butyrate and Zn^2+^, as in A, in the presence of the PI3 kinase inhibitor (wortmannin). **C.** Averaged rates of pH_i_ recovery following addition of Na^+^ as determined from the traces. n = 3; *p<0.05. **D.** Cell survival of HT29 colonocytes was measured using the SRB assay. Cells were treated with butyrate and Zn^2+^, as in Figure 1, in the presence or absence of 0.5 µM cariporide. n = 3; *p<0.05.

Butyrate induces an acid load and triggers activation of NHE [41]. To determine whether Zn^2+^ enhances the rate of recovery of intracellular pH we monitored changes of BCECF fluorescence in butyrate treated HT29 cells. Application of 30 mM butyrate in nominally Na^+^-free Ringer's solution, resulted in acidification of the cells (Fig. 5A). Addition of Na^+^ to the Ringer's solution was followed by slow recovery of pHi, consistent with a role for NHE in reducing the acid load triggered by butyrate [42]. Rates of pH_i_ recovery were compared between cells pretreated with Zn^2+^, applied at concentration and duration sufficient to activate the ZnR, and controls (Zn^2+^-free Ringer's solution). As shown in Figure 5A, the rate of recovery was 7.1±0.01 fold higher in Zn^2+^ pretreated cells. To determine if pHi recovery is mediated by NHE1, the major isoform expressed in HT29 cells, we applied the NHE1 inhibitor cariporide (0.5 µM). This was followed by a block of both the Zn^2+^-dependent and basal pH_i_ recovery, indicating that cellular pHi recovery is primarily mediated by NHE1 in HT29 cells. We then asked if the signaling pathways activated by ZnR are linked to the enhanced pHi recovery from butyrate acid load. The rate of pHi recovery in cells that were pre-treated with wortmannin (30 nM) prior to application of Zn^2+^ was attenuated compared to the rate monitored in control cells (Fig. 5B–C). This set of experiments indicates that ZnR activation via PI3 kinase mediates Zn^2+^-dependent upregulation of NHE activity.

Next we asked if Zn^2+^ promotes cell survival by accelerating the pH_i_ recovery through activation of the NHE. Cells were treated with the NHE1 inhibitor cariporide (0.5 µm), which completely blocked the recovery from butyrate-induced acid load (Fig. 5C), then butyrate and Zn^2+^ were applied (as described in Fig. 1) and cell survival was determined. Cell numbers following butyrate and Zn^2+^ treatment were similar in the presence or absence of cariporide (Fig. 5D), suggesting that a different downstream protein, and not NHE1 is linked to the pro-survival effects of Zn^2+^ on HT29 cells.

### Clusterin (CLU) is up-regulated by Zn^2+^ acting via ZnR

**Figure 6:**
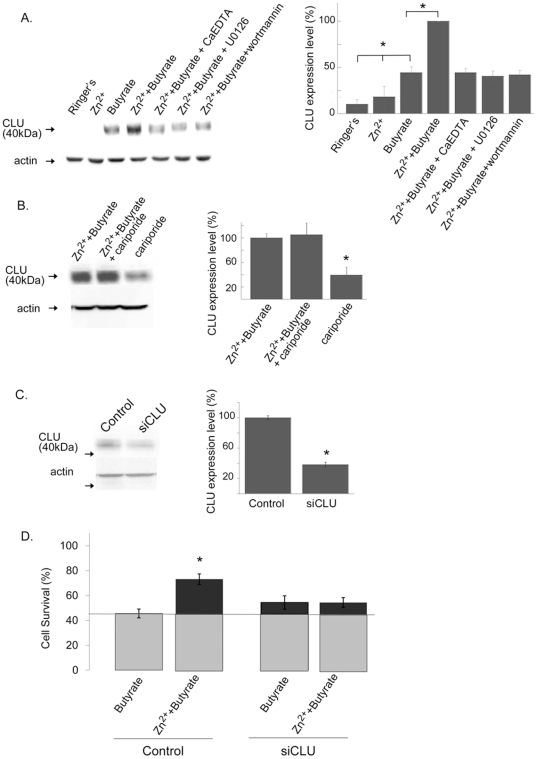
Zn^2+^ and butyrate upregulate the expression of the pro-survival protein clusterin (CLU). **A.** *left panel*: Immunoblot analysis using anti α-CLU antibodies was done on lysates from HT29 cells treated with either Zn^2+^, butyrate or both, in the presence or absence of the cell impermeable Zn^2+^ chelator CaEDTA, or the kinase inhibitors as indicated. *right panel*: Densitometeric analysis of α-CLU expression. n = 3; *p<0.05. **B.** *left panel:* Immunoblot of cell lysates from HT29 cells treated as in A in the presence or absence of cariporide (0.5 µM). *right panel*: Densitometeric analysis of α-CLU expression. n = 3 *p<0.05. **C.** CLU expression was monitored using westernblot analysis in HT29 cells transfected with an siCLU or scrambled siRNA (Control) constructs. Using prolonged exposure time (180 s) CLU expression could be monitored in these cells. Actin was used as a loading control. *Right panel*: Densitometry analysis of CLU expression level in control and siCLU transfected cells. **D.** Cells were transfected with an siCLU construct and treated as in Figure 1, cell numbers were monitored using the SRB colorimetric assay and compared to control cells. n = 3, *p<0.05.

The pro-survival protein clusterin (CLU) plays an important role in promoting tumor cells proliferation and serves as a marker for colon cancer [43]. We therefore studied the effect of butyrate and Zn^2+^ on CLU expression in colonocytes. HT29 cells were treated with butyrate with or without Zn^2+^, as described in Figure 1 (see also Materials and Methods). When applied alone, Zn^2+^ did not enhance CLU expression but butyrate induced an increase in CLU expression compared to control cells (Fig. 6A). Treatment with Zn^2+^ and butyrate was followed by a further increase in CLU expression level (55±12% increase) compared to treatment with butyrate only. Application of the cell impermeable Zn^2+^ chelator, CaEDTA (100 µM), in the presence of Zn^2+^ and butyrate reversed the increase of CLU expression, indicating that the rise in CLU levels is induced by extracellular Zn^2+^. We then sought to determine the role of ZnR signaling in mediating the effects of Zn^2+^, using the MAPK (U0126, 1 µM) or PI3K (wortmannin, 30 nM) inhibitors. Application of each of these inhibitors eliminated the stimulatory effect of Zn^2+^ on CLU expression (Fig. 6A). These results indicate that ZnR signaling is essential for Zn^2+^-dependent upregulation of CLU expression. To determine if CLU expression is modulated by ZnR-dependent activation of NHE1, HT29 cells were treated with Zn^2+^ and butyrate in the presence or absence of the NHE1 inhibitor. Cariporide (0.5 µm), applied at a concentration that blocked ZnR-dependent activation of NHE1 (Fig. 5C), did not affect the Zn^2+^-dependent upregulation of CLU expression (Fig. 6B). Thus, Zn^2+^ regulates CLU expression via activation of ZnR signaling but independent of NHE1.

We next studied the role of CLU in mediating the ZnR-dependent enhanced cell survival, HT29 cells were transfected with an siRNA construct targeted to silence CLU expression (siCLU). To assess the efficiency of CLU silencing we used western blot analysis. Since basal levels of CLU monitored following prolonged starvation in the non-transfected HT29 cells were low (Fig. 6A) we treated transfected HT29 cells with serum-containing medium for 24 h. Under these conditions CLU expression was apparent and was significantly reduced by the siCLU (38±3% of siCont, Fig. 6C). Survival rates were then determined in the siCLU transfected cells following butyrate and Zn^2+^ treatment. Following exposure to butyrate, survival rates of siCLU transfected cells were similar to those of the control cells (54±9% and 45±10%, respectively, Fig. 6D). However, no Zn^2+^-dependent enhancement of the survival was monitored in the siCLU transfected cells (54±7%). This indicates that Zn^2+^ is inducing the pro-survival effect on HT29 cells via upregulation of clusterin expression.

**Figure 7:**
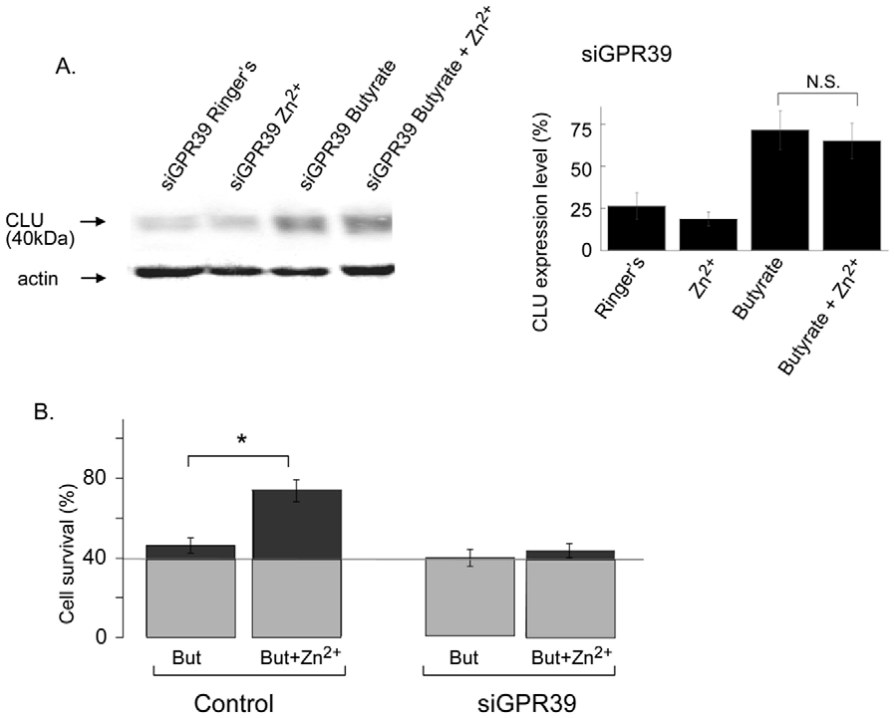
GPR39/ZnR mediates Zn^2+^-dependent activation of the pro-survival protein, CLU, and rescues cells from butyrate induced cell death. **A.** HT29 cells transfected with an siGPR39 construct, were treated with butyrate or without it (Ringer's solution alone), in the presence or absence of Zn^2+^ (as described in Fig. 1). Cell lysates were subjected to immunoblotting with an anti-α-CLU, actin levels are presented as control. Densitometry analysis of the results is shown in the *right panel*, normalized to anti-α-CLU expression level in control (non-transfected) cells treated with Ringer's solution (100%). n = 3. **B.** The siGPR39 cells or controls were treated with butyrate with or without Zn^2+^ as described in Figure 1. Cells were then fixed and number of cells was monitored using the SRB colorimetric assay, gray line indicates cell numbers in butyrate only treated siGPR39-transfected cells. n = 3; *p<0.05.

Finally, we sought to determine if GPR39 is the molecular moiety which mediates the pro-survival effects of ZnR. We first studied the effect of GPR39 silencing on the Zn^2+^-dependent upregulation of CLU levels following butyrate treatment. Transfection with the siRNA somewhat increased the basal CLU level in control cells treated with Ringer's solution only (Fig. 7A). In the siGPR39 transfected cells, similar to non-transfected cells, butyrate treatment upregulated CLU expression compared to the control cells (Fig. 7A). In contrast to the non-transfected cells, Zn^2+^ did not increase CLU expression further in the siGPR39 transfected cells (Fig. 7A). This result indicates that the effect of Zn^2+^ on upregulation of CLU is mediated by ZnR/GPR39. Next, we studied the effect of silencing GPR39 expression on survival of HT29 cells. Zn^2+^ rescued HT29 cells transfected with a control siRNA from butyrate induced cell death (Fig. 7B), similar to its effect on the non-transfected HT29 cells (Fig. 1A). Exposure of the siGPR39-transfected cells to butyrate with or without Zn^2+^ treatment resulted in similarly low cell survival, 42±7% and 39±10% (respectively) of control cells not treated with butyrate (Fig. 7B). Altogether, our data indicate that GPR39 is the ZnR, and is mediating the effect of Zn^2+^ on CLU expression leading to enhanced cell survival.

## Discussion

A role for extracellular Zn^2+^ in activating major signaling pathways linked to cellular proliferation and survival has been previously demonstrated [44]. The aim of this study was to identify the molecular moiety responsible for Zn^2+^ signaling and determine the physiological role of this pathway in promoting survival in the unique acidic environment of the colon. Our results strongly indicate that GPR39 is triggering ZnR signaling in colonocytes and HT29 cells, as silencing of GPR39 completely abolished the Zn^2+^-dependent intracellular calcium release a hallmark of ZnR activity. Interestingly, silencing of GPR39 was followed by a decrease in basal cell growth, suggesting that even residual Zn^2+^ in the medium, via ZnR/GPR39, is sufficient to enhance cell growth. Also, profound ZnR-dependent enhancement of NHE activity in colonocytes obtained from WT mice was virtually eliminated in colon tissue obtained from GPR39 KO mice. NHE transporters are the major Na^+^ influx pathway in the colon thereby regulating solute transport [41], [45], [46], it will therefore be interesting to study if the acute regulation of this pathway by Zn^2+^ via ZnR/GPR39 may be linked to the antidiarrheal effects of zinc. Finally, our results indicate that ZnR/GPR39 activation enhances survival of colon cancer cells by rendering them less susceptible to cell death induced by butyrate.

The presence of high concentration of SCFAs in the colon, among them butyrate, generates a unique homeostatic environment that is essential for colonocytes nutrition but imposes an acidic stress on these cells. Many studies have indicated that butyrate is a major factor in selective colon tumor cell death, acting via regulation of cell-cycle related proteins [47], [48]. We show that ZnR signaling affects the resistance of HT29 cells to butyrate induced cell death. Our results indicate that Zn^2+^-dependent activation of the ZnR rescues HT29 colonocytes from butyrate treatment. Butyrate has been suggested to reduce colon cancer cell survival by inhibiting histone deacetylase activity [49]. Since histone deacetylase is a Zn^2+^-dependent protein [50] it may be argued that Zn^2+^ can directly interfere with inhibition of histone deacetylase by butyrate and thereby reverse the activity of butyrate on cell survival. Our results do not support such scenario since Zn^2+^ permeation into colonocytes at the time intervals used to trigger ZnR activity is minimal [22]. Hence it is unlikely that Zn^2+^, as applied in this study, affected histone deacytalse activity but it was sufficient to enhance HT29 colonocyte survival. Moreoever, molecular silencing of ZnR/GPR39 or desensitization of the receptor reversed the protective effect of Zn^2+^. Furthermore, inhibition of the MAP and PI3 kinase pathways which are activated by Zn^2+^ via ZnR/GPR39 [22] also reversed the protective effect of Zn^2+^. These results are also consistent with the ZnR-dependent enhanced prostate cancer cell survival that we have shown previously [21].

Based on the key role of Na^+^/H^+^ exchange in regulating pHi and reducing acid load, we reasoned that this pathway may be involved in the rescue of HT29 colonocytes from butyrate. Inhibition of NHE indeed abolished the ZnR-dependent enhanced pH_i_ recovery from the acid load induced by butyrate. Despite the documented role of NHE in cell survival [51], [52] the Zn^2+^-dependent activation of NHE did not enhance survival rates of HT29 cells following administration of butyrate, indicating that a distinct mechanism is mediating the effect of Zn^2+^ on cell survival. It should be noted that colonocytes are chronically exposed to SCFA-induced acid load that can overwhelm the ability of NHE to trigger effective pHi recovery and thus these cells may have a different mechanism to enhance cell survival. We have previously shown that prolonged acidification, independently of Zn^2+^, upregulates NHE activity [22] and may regulate intracellular pH during the prolonged acid load. In accordance, butyrate may exert a pro-apoptotic effect that is independent of the acidification [53], [54], and thus ZnR-dependent activation of anti-apoptotic pathways will be a critical event for the rescue of the cells by zinc. Indeed, we show that Zn^2+^, via ZnR/GPR39, induces expression of clusterin in cells exposed to butyrate. Clusterin has a well-documented anti-apoptotic effect, which is of clinical importance in enhancing the resistance of tumor cells to a large variety of pro-apoptotic stimuli [55]. Clusterin expression was linked both to cell death or survival, numerous studies indicate that the nuclear (nCLU, 65 kDa) or the secreted (sCLU, 40 kDa) isoforms of this protein have distinct roles, such that sCLU is linked to cell survival while nCLU is associated with cell death [56]. Hence, the increased expression of sCLU induced by Zn^2+^ suggests that this pathway is enhancing cell survival. Inhibition of Zn^2+^-dependent clusterin induction by MEK inhibitors further supports an anti-apoptotic effect for Zn^2+^. This pathway participates, for example, in enhancing a pro-survival effect following ionizing radiation treatment via IGF-1R activation [57]. Increase in secreted clusterin levels, triggered via the PI3K pathway activation, was associated with chemoresistance of prostate cancer cells [58]. Consistent with this effect, our results show that ZnR-dependent sCLU expression requires activation of AKT, further suggesting a survival role for the ZnR-dependent pathway. While extracellular Zn^2+^ via the ZnR is shown to promote cell survival, an opposing role is suggested for intracellular Zn^2+^ rise that often leads to cell death [59], [60], [61], [62], [63]. Interestingly, upregulation of the pro-apoptotic nCLU form by ischemia, which is known to induce a rise in intracellular Zn^2+^, has been shown to play a role in neuronal death [64], [65]. Consistent with such a role, intracellular Zn^2+^ or Cd^2+^ rise induces expression of the pro-apoptotic nCLU and triggers testicular germ cells death [66]. Interestingly, previous works have also suggested that intracellular Zn^2+^ rise following prolonged exposure to this ion, may result in colon cancer cell death [67]. Thus, we suggest that while intracellular Zn^2+^ enhances the expression of nCLU and induces cell death, extracellular Zn^2+^ activates the ZnR/GPR39 signaling pathway and induces the expression of sCLU leading to enhanced survival.

In conclusion, our data indicate the ZnR links between Zn^2+^ and clusterin, a major player in colonocyte survival or apoptosis [68]. By enhancing clusterin expression Zn^2+^, via the ZnR, renders HT29 cells less susceptible to pro-apoptotic agents in the digestive tract and may be of importance in digestive system function and disease.
